# Association of vitamin D, oxidative stress markers, and hormonal factors with male infertility: a case-control study in Pakistan

**DOI:** 10.55730/1300-0144.6217

**Published:** 2026-01-29

**Authors:** Muhammad TAYYAB IHSAN, Ayesha ALI MALICK, Sher KHAN, Muhammad TAHIR KHAN, Haq NAWAZ KHAN, Rehana REHMAN

**Affiliations:** 1 Biological and Biomedical Sciences, The Aga Khan University, Stadium Road Karachi, Pakistan; 2 Department of Biochemistry, Federal Urdu University of Arts, Science and Technology, Karachi, Pakistan; 3 Department of Biotechnology, University of Karachi, Pakistan; 4 Department of Pathology, The Aga Khan University, Karachi, Pakistan

**Keywords:** Male infertility, oxidative stress, vitamin D

## Abstract

**Background/aim:**

Infertility impacts around 15% of couples worldwide, with male factors accounting for approximately 50% of cases. Despite this significant share, male infertility remains understudied, particularly in developing nations such as Pakistan. The present study explores the association between male infertility and seminal plasma concentrations of oxidative stress markers, inhibin B, and vitamin D, along with their relationship with sperm parameters.

**Materials and methods:**

The dataset of the study consisted of seminal plasma levels from 30 fertile and 60 infertile males. Total antioxidant capacity (TAC), Vitamin D, malondialdehyde (MDA), 4-hydroxynonenal (4-HNE), lipid peroxide (LPO), superoxide dismutase (SOD), and inhibin B levels were assessed using ELISA kits and the data were analyzed using SPSS (v.23) software. Descriptive statistics for quantitative variables were reported as mean ± standard deviation. Correlations among parameters were assessed using Spearman’s rank correlation test. In all analyses, p-values of < 0.05 were considered significant, and < 0.001 as highly significant.

**Results:**

Fertile males exhibited higher vitamin D and TAC levels than the infertile males (p-values = 0.00 and < 0.01, respectively). In contrast, higher MDA and 4-HNE levels were observed in infertile males (p-value = 0.00). No statistically significant relationship was identified between the inhibin B and LPO values of the two groups.

**Conclusion:**

Elevated oxidative stress markers (MDA, 4-HNE) and reduced vitamin D and TAC levels were associated with infertility in the sample. The dual role of SOD in oxidative stress regulation warrants further investigation to determine the thresholds at which sperm integrity is affected. Vitamin D may play a critical function in spermatogenesis, though its exact mechanisms require further exploration. These findings underscore the role of oxidative stress and nutritional factors in fertility, and highlight the need for targeted interventions to improve reproductive outcomes.

## Graphical Absctract

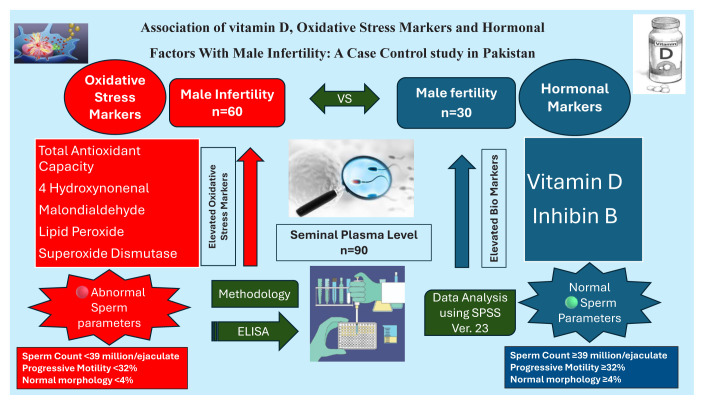

## 1. Introduction

Infertility affects approximately 15% of couples worldwide, with male factors accounting for around 50% of cases [1]. Male infertility (MI) is defined as the inability of a sexually active couple to achieve conception after 12 months of regular, unprotected intercourse when the cause is attributable to a problem in the male partner [2]. According to the World Health Organization, 180 million people are affected, and the burden is particularly significant in developing countries such as Pakistan, where the prevalence of infertility is estimated to be around 22% [2]. Despite the high burden, MI remains under-investigated, especially in South Asian populations, where cultural and social stigma often obscure its recognition and management. The limited understanding of male infertility mechanisms in this region highlights the need for research into novel and accessible biomarkers [3].

MI has attracted little academic interest to date, and while there have been studies reporting a greater prevalence of the condition in developed countries than in underdeveloped countries, there is a dearth of data on the reasons for infertility and the available treatment options [4].

Among the various regulators of male fertility, hormones play a critical role in spermatogenesis and testicular function [5]. Inhibin B, secreted by Sertoli cells, is a well-recognized biomarker of spermatogenic activity and has been correlated with sperm concentrations and testicular function [ 6 ]. Likewise, vitamin D has gained interest as a potential regulator of male reproductive health. Beyond its role in calcium homeostasis, vitamin D receptors and metabolizing enzymes are expressed in the testes, epididymis, prostate, and spermatozoa, suggesting possible local actions in sperm maturation and motility [ 6,7 ]. However, the evidence remains inconclusive, and most studies rely on serum vitamin D, while the potential relevance of seminal vitamin D concentrations is less explored [8].

Oxidative stress is another major contributor to male infertility. Reactive oxygen species (ROS), when present in physiological concentrations, support such essential sperm functions as capacitation and acrosome reaction [9], while excessive production can lead to lipid peroxidation, DNA damage, and impaired sperm function. Biomarkers such as malondialdehyde (MDA) and 4-hydroxynonenal (4-HNE) indicate lipid peroxidation, while total antioxidant capacity (TAC) and enzymes like superoxide dismutase (SOD) reflect the antioxidant defense status [ 10 ]. An imbalance between these systems has been consistently associated with poor semen quality and infertility. Yet, little is known about the interplay between seminal vitamin D and oxidative stress markers in infertile men [ 11 ].

Given the high prevalence of male infertility and the lack of data on the condition in Pakistan, there is a pressing need to identify reliable and non-invasive biomarkers [ 12 ]. In contrast to the earlier studies in which the focus was primarily on serum parameters, the present study evaluates seminal plasma concentrations of vitamin D, oxidative stress markers, and inhibin in fertile and infertile men [ 13 ].

### 1.1. Study rationale and hypotheses

There have been few studies to date examining vitamin D, oxidative stress markers, and inhibin simultaneously in seminal plasma, particularly in South Asian populations [ 16 ]. Methodologically, this study differs from most earlier research in which the focus has been on serum biomarkers, which may not fully explain the reproductive microenvironment [ 17,18 ]. Biologically, the study considers both hormonal and oxidative stress markers together to capture their combined impact on male fertility. Population-specific novelty is provided by analyzing seminal plasma from fertile and infertile men in Pakistan, generating evidence related to this understudied group [19]. We hypothesize that infertile men will have lower levels of seminal vitamin D and TAC, and higher levels of oxidative stress markers (MDA, 4-HNE, LPO, and SOD) than fertile men. Furthermore, we hypothesize that seminal vitamin D will be positively correlated with sperm count, motility, and morphology, and negatively correlated with noninvasive oxidative stress markers.

## 2. Materials and methods

The study was conducted in the Multidisciplinary Lab, Biological & Biomedical Sciences, The Aga Khan University, Karachi (AKU) from June 2022 to April 2023. Ethical approval for the study was granted by the AKU Ethics Review Committee (ERC No. 4813- BBS- ERC-17), and was carried out in accordance with the principles of the Declaration of Helsinki. A total of 90 seminal plasma samples were acquired for the study from donors aged 18–35 years, including 30 with normal sperm parameters and 60 with abnormal sperm parameters. The sperm parameter standards adopted for the study were the World Health Organization (WHO) reference values for human semen (fifth edition, published in 2010), with sperm count ≥39 million/ejaculate, progressive motility ≥32%, and normal morphology ≥4% being the lower reference limits. Men with normal semen parameters were defined as fertile, while those with abnormal semen parameters were defined as infertile. Paternity status was not recorded, which can be considered a limitation of the study. The samples were collected in sterile 15 mL bottles by the donors, acquired through masturbation in a private setting, at the Australian Concept Infertility Center (ACIMC), and all provided consent for their inclusion in the study. A detailed semen analysis report (SAR) of the samples was compiled by the ACIMC, and the samples were transferred to AKU and kept in a temperature-controlled environment. The average time between the sample processing at the ACIMC and the arrival of the samples at AKU was 35 minutes. Upon arrival at AKU, the seminal samples were centrifuged at 16 000 × g at 4 ºC to isolate seminal plasma, which was then aliquoted into 1.5 mL microtubes.

The samples were analyzed using an enzyme-linked immunosorbent assay (ELISA) to assess TAC (Cat. No. E-2199 Hu, with a standard curve range of 0.3–90 U/mL and a sensitivity of 0.140 U/mL), vitamin D (Vit D, Cat. No: EA0057Hu, Human 1, 25-dihydroxyvitamin D3, DVD, DHVD3 ELISA Kit), MDA levels (Cat. No E1371Hu, standard curve range: 0.2–70 nmol/mL, sensitivity: 0.14 nmol/mL), hydroxynonenal (4-HNE, Cat. No: EA0066Hu, standard curve range: 0.2–70 nmol/mL, sensitivity: 0.14 nmol/mL), lipid peroxide (LPO, Cat. No E4437Hu, standard curve range 0.05–9 ng/mL, sensitivity 0.023 ng/mL), SOD (Cat. No E4502Hu, standard curve range 0.5–180 ng/mL, sensitivity 0.24 ng/mL), and inhibin B (Cat. No: EA0020Hu, standard curve range 7–1500 ng/L, sensitivity: 3.22 ng/L) in accordance with the kit manufacturers’ instructions. The participants were grouped as fertile or infertile, according to the WHO 2010 semen parameter criteria. For the exploratory analysis, the participants were further stratified post hoc into vitamin D status categories (deficient, insufficient, sufficient) based on Endocrine Society guidelines to identify associations between vitamin D levels and semen/biochemical parameters [46].

The study parameter concentrations, determined by ELISA, were statistically analyzed using SPSS version 23 (IBM Corp., Armonk, NY, USA). Descriptive statistics for quantitative variables were expressed as mean ± standard deviation (Mean ± SD). Between-group comparisons were conducted using independent t-tests, and correlations were assessed using Spearman’s rank correlation coefficient. Due to the relatively small fertile group (n = 30), no multivariable logistic regression was performed to avoid model overfitting. Statistical significance was set at p < 0.05, with p < 0.001 considered highly significant. Lifestyle and clinical confounders such as smoking, alcohol intake, occupational exposure, and varicocele were not systematically collected.

## 3. Results

The clinical characteristics, semen parameters, and biochemical markers of the fertile and infertile groups are summarized in Table 1. The mean age in the fertile group was slightly higher than that of the infertile group (37.53 ± 5.67 vs 34.60 ± 6.52 years, p = 0.04) while the body-mass index was lower (25.07 ± 2.75 vs 27.25 ± 4.12 kg/m^2^, p = 0.01). Sperm morphology, motility, and count were all significantly higher in the fertile group (16.57 ± 1.74% vs 1.38 ± 1.17%; 28.52 ± 18.73% vs 6.59 ± 4.32%; 56.15 ± 36.76 vs 10.75 ± 7.93 million/mL, respectively, all p < 0.001). Seminal vitamin D levels were higher in the fertile group (34.12 ± 9.66 vs 23.98 ± 10.77 fmol/L, p < 0.001), whereas markers of oxidative stress were higher in the infertile group, including MDA (4.11 ± 2.63 nmol/mL vs 11.46 ± 4.59 nmol/mL, p < 0.001) and 4-HNE (0.53 ± 0.35 vs 1.63 ± 0.63 ng/L, p < 0.001). There was no significant difference in LPO (p = 0.27); SOD was higher in the infertile group (79.31 ± 33.46 vs 106.73 ± 57.87 ng/mL, p = 0.02), while TAC was lower (3.55 ± 1.03 vs 2.74 ± 0.78 U/mL, p < 0.01). There was no significant difference between the groups in inhibin B levels (p = 0.23).

Correlations between vitamin D and the study parameters are presented in Table 2. Vitamin D was positively associated with sperm morphology (r = 0.50, p < 0.01), motility (r = 0.33, p < 0.01), count (r = 0.24, p = 0.02), and TAC (r = 0.72, p < 0.01), and negatively with MDA (r = −0.32, p < 0.01), 4-HNE (r = −0.28, p = 0.01), and SOD (r = −0.28, p = 0.01), while weaker but still significant associations were observed with sperm count and 4-HNE (p < 0.05). No significant correlations were observed with age, BMI, lipid peroxide, or inhibin B.

The associations of sperm parameters with oxidative stress markers are presented in Table 3. Sperm morphology, motility, and count were negatively correlated with MDA (r = −0.63, −0.45, and −0.46, all p < 0.001) and 4-HNE (r = −0.68, −0.46, and −0.48, all p < 0.001). TAC was positively correlated with sperm morphology (r = 0.43, p < 0.001) and sperm count (r = 0.21, p = 0.04), while correlations with LPO and inhibin B were not significant. Vitamin D correlated with sperm morphology (r=0.50, p < 0.001) and sperm motility (r=0.33, p < 0.001), to a highly significant degree.

The differences across vitamin D categories are presented in Table 4. Participants with sufficient vitamin D levels exhibited higher sperm morphology (9.49 ± 7.55% vs 1.82 ± 4.10% in the deficient group, p < 0.01), motility (17.21 ± 15.47% vs 6.34 ± 4.51%, p < 0.05), and count (29.92 ± 31.40 vs 10.35 ± 6.87 million/mL, p < 0.01), lower MDA (7.48 ± 4.35 vs 11.58 ± 5.70 nmol/mL, p = 0.01) and 4-HNE (1.18 ± 0.81 vs 1.60 ± 0.67 ng/L, p = 0.01), and higher TAC (3.69 ± 0.79 vs 2.29 ± 0.63 U/mL, p < 0.001). SOD showed a borderline difference (p = 0.05), while age, BMI, lipid peroxide, and inhibin B did not vary significantly across the vitamin D categories.

## 4. Discussion

This study examined the association of seminal vitamin D, oxidative stress (OS) markers, and inhibin B with male infertility in a Pakistani cohort. The infertile participants exhibited significantly higher levels of MDA and 4-HNE and lower TAC, while vitamin D concentrations were significantly higher in the fertile group. Vitamin D was positively associated with sperm count, motility, and morphology, and negatively associated with MDA and 4-HNE. These findings indicate an association between lower vitamin D status and increased OS in male infertility, consistent with several previous reports [20,23].

Vitamin D is primarily recognized as a hormonal factor, although there is emerging evidence suggesting that it may also modulate redox balance. Proposed mechanisms include regulation of intracellular calcium flux, trans-epithelial calcium transport in the epididymis, and modulation of mitochondrial reactive oxygen species generation [20]. Consequently, the role of vitamin D in male infertility remains unconfirmed, indicating a need for further study, although it is believed to play a role in spermatogenesis and spermatozoa maturation. Enzymes associated with vitamin D receptors have been identified in different locations, including the prostate, germ cells, seminal vesicles, mature spermatozoa, and epididymis [22]. Several in vitro studies to date have suggested that vitamin D is involved in the transfer of trans-epithelial calcium within the epididymis [24]. Calcium is essential for spermatogenesis and the acrosome reaction, for which 25(OH)2D3 serves as a key regulator [25]. Other proposed mechanisms influencing increased sperm motility involve adenosine-triphosphate synthesis, mediated by both elevated cyclic-adenosine monophosphate levels and the activation of the protein kinase A pathway. Although our data demonstrate significant relationships, the cross-sectional study design precludes causal inference, and vitamin D may be associated with, but cannot be concluded to directly influence, spermatogenesis [26].

The production of reactive species persists even after the cellular metabolism has returned to normal [27]. OS has a profound impact on the production of defective sperm parameters – one of the major causes of male infertility [28]. Our study revealed MDA and 4-HNE to be elevated in infertile males, which can be considered a significant finding [22]. Specifically, numerous links have been established between 4-HNE and its effects on sperm motility and morphology, as well as the interaction of the acrosomal head and the zona pellucida of oocytes [29,30]. The exposure of biological molecules to 4-HNE has been shown to induce an increase in the production of mitochondrial ROS, resulting in a cascade of oxidative stress in the spermatozoa. 4-HNE can disrupt a varied array of cellular functions, being one of the most physiologically active lipids [30]. 4-HNE levels were noted to be higher in the infertile group in the present study, consistent with previous studies, and negatively impacted sperm motility and morphology [13,31]. The results of our analysis of the association between MDA levels and infertility also concur with previous studies reporting elevated MDA in oligozoospermia when compared to normozoospermic groups [32,33]. MDA has numerous negative impacts on sperm viability and sperm motility [28,34]. The effect on sperm concentrations is still open to debate, as some studies report elevated MDA concentrations with reduced sperm concentrations, while others report the opposite [35, 36]. Although MDA has been proposed as a biomarker of semen quality, its elevation in various inflammatory conditions, such as varicocele and infections, should be kept in mind [ 35 ]. That said, MDA can help identify which pathologies result in altered sperm parameters. Elevated MDA and 4-HNE levels in infertile men indicate enhanced lipid peroxidation, and these markers have been linked to impaired sperm motility and morphology in previous studies [29, 33]. 4-HNE, a highly reactive aldehyde, can disrupt the sperm-oocyte interaction and induce mitochondrial ROS production, amplifying oxidative damage [30]. MDA, likewise, has been proposed as a marker of semen quality, but may also be elevated in the presence of such inflammatory conditions as varicocele and infection, which were not systematically assessed in the present study [ 36 ].

Positive correlations between sperm count and motility with SOD levels, and negative correlations with sperm DNA fragmentation and SOD levels have been reported in previous literature, which is a biologically reasonable finding [ 37 ]. The findings of the present study contradict most in the existing literature, as higher SOD levels were observed in the infertile group than in the fertile group. This difference may be attributed to the different levels of SOD reported with different effects in previous studies, and suggestions that higher SOD levels than necessary may actually be toxic to spermatozoa [ 38 ]. Elevations in antioxidant levels increase the fluidity of the plasma membrane, and subsequently, the potential for acrosomal damage [ 39 ]. Together, these observations highlight the complexity of redox regulation in the male reproductive tract. SOD overexpression, therefore, may reflect impaired sperm maturation, leading to reduced sperm fertilization capacity [ 38 ].

TAC is the measure of the body’s ability to counteract OS. It is an indirect measure of OS, and has been shown to be reduced in infertile semen samples [ 17 ]. This is confirmed by the present study, in which TAC levels were significantly lower in the infertile group. Earlier studies have discussed the contribution of TAC to the pathophysiology of infertility, irrespective of diagnosis, with low levels being directly correlated with reduced sperm concentration, motility, and morphology. Whether TAC levels are indicative of OS depends largely on the source of TAC, that is, whether it is produced intracellularly or extracellularly [ 40 ]. ROS scavengers such as catalase and SOD are relatively ineffective when ROS are produced intracellularly. In cases where ROS are produced extracellularly, TAC may be a viable biomarker of the extent of OS [ 41 ]. In contrast to the significant differences observed for MDA, 4-HNE, SOD, and TAC; there was little difference in the LPO and inhibin B values observed for the two groups. This finding aligns with earlier studies suggesting that LPO may be a less sensitive indicator of OS in seminal plasma than MDA and 4-HNE.

Inhibin B is a well-known biomarker of Sertoli cell activity that reflects spermatogenesis, although its ability to reflect sperm concentrations may show limited variation in case-control designs when group definitions are based primarily on semen parameters. These non-significant results may also be attributed to biological variability or the modest sample size, and should, therefore, be interpreted with caution [ 42 ]. No significant association related to inhibin levels was noted in either the fertile or infertile groups, or between inhibin levels and vitamin D. Vitamin D, when compared to several biomarkers, had a weak negative correlation with two oxidative stress markers: MDA and 4-HNE, and a strong significant association with TAC. The relationship between vitamin D and MDA is supported by previous studies [44,45]. Norouzabad observed that vitamin D, when taken orally, altered MDA seminal levels in infertile men and resulted in variations in vitamin D concentrations [ 43 ]. With regards to 4-HNE, significant negative correlations have been reported following vitamin D supplementation [12,45].

In the present study, the infertile group had higher BMI values and were slightly older than the fertile controls, and both of these factors can affect oxidative balance and hormone concentrations, and may account in part for some of the observed associations. Lifestyle factors such as smoking, alcohol consumption, occupational exposures, and varicocele were not systematically recorded in the present study, and so could not be included as covariates in the analyses. The results of the present study are summarized in Figure.

## 5. Conclusion

In the present study, significantly lower seminal vitamin D and TAC values were observed in the infertile group, alongside higher MDA and 4-HNE levels, when compared to the fertile group. These findings reinforce the role of OS and nutritional factors in MI. Interestingly, SOD levels were noted to be elevated in the infertile group, which may be indicative of a compensatory but potentially harmful antioxidant response. No significant associations were found with LPO and inhibin, suggesting these biomarkers may have limited diagnostic utility in seminal plasma. Overall, the study highlights the potential of seminal biomarkers, particularly vitamin D and OS markers, as non-invasive tools that can contribute to a better understanding of male infertility in Pakistan.

### Limitations and Future Recommendations

This study has several limitations that should be acknowledged. First, the case-control design precludes the establishment of causal relationships between biomarkers and infertility. Second, as mentioned above, important confounding factors such as smoking, alcohol use, varicocele, and occupational exposures were not systematically assessed, and BMI and age differences between groups may have influenced the results. Additionally, as no multivariable adjustment was performed, the potential confounding effects of these variables on the observed associations cannot be excluded, and the findings should thus be interpreted with caution. Third, the sample size was relatively modest (n = 90), limiting the statistical power of the study to detect smaller associations. A power analysis indicated that the sample size (n = 90) provides approximately 80% power to detect medium–large group effect sizes, but may lack the power for the detection of smaller effects. Fourth, seminal vitamin D assays are less standardized than serum measurements, limiting comparisons with previous studies. Moreover, fertility status was classified based solely on WHO 2010 reference values, and paternity was not verified, which may limit the certainty of the group classification. Despite these limitations, the study provides novel insights into the relationship between seminal vitamin D, oxidative stress, and MI. Finally, vitamin D was quantified in fmol/L using an ELISA kit, and so the category cut-offs reflect the manufacturer’s reference ranges, which are not directly comparable to the serum-based ng/mL thresholds recommended by the Endocrine Society. This limits the direct comparison of the study findings with other studies reporting serum vitamin D status.

Among its strengths, the study adds to the limited available data through its evaluation of seminal rather than serum biomarkers in infertile men from Pakistan, thereby providing population-specific insights that are directly relevant to this understudied population within South Asia. Further larger, well-controlled studies are warranted to validate the findings of this study and to explore the clinical applications.

## Figures and Tables

**Figure:**
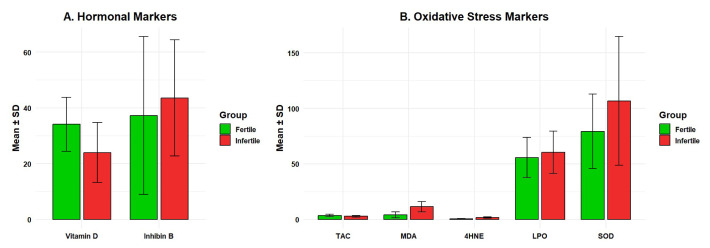
Comparison of seminal vitamin D, hormonal, and oxidative stress biomarkers (mean ± SD) between fertile and infertile men.

**Table 1:** Comparison of clinical characteristics, semen parameters, and biochemical markers between fertile and infertile males.

Variables	Fertile males (n=30)	Infertile males (n=60)	p-value
**Age (Years)**	37.53 ± 5.67	34.6 ± 6.52	0.04
**Body mass index (kg/m** **^2^** **)**	25.07 ± 2.75	27.25 ± 4.12	0.01
**Sperm morphology (%)**	16.57 ± 1.74	1.38 ± 1.17	p < 0.00
**Sperm motility (%)**	28.52 ± 18.73	6.59 ± 4.32	p < 0.00
**Sperm count (million/mL)**	56.15 ± 36.76	10.75 ± 7.93	p < 0.00
**Vitamin D (fmol/L)**	34.12 ± 9.66	23.98 ± 10.77	p < 0.00
**Malondialdehyde (nmol/mL)**	4.11 ± 2.63	11.46 ± 4.59	p < 0.00
**4-Hydroxynonenal (ng/L)**	0.53 ± 0.35	1.63 ± 0.63	p < 0.00
**Lipidperoxide (ng/mL)**	55.74 ± 18.12	60.35 ± 18.99	0.27
**Superoxidedismutase (ng/mL)**	79.31 ± 33.46	106.73 ± 57.87	0.02
**Inhibin B (ng/L)**	37.27 ± 28.26	43.57 ± 20.8	0.23
**Total antioxidant capacity (U/mL)**	3.55 ± 1.03	2.74 ± 0.78	< 0.01

The stratification into normal and abnormal sperm parameters was based on WHO guidelines for semen analysis. p < 0.05 was considered significant; p < 0.001 was considered highly significant.

**Table 2:** Correlation of seminal vitamin D with clinical, semen, and biochemical parameters.

Variable	Vitamin D (fmol/L)	
	r	p-value
**Age (years)**	0.15	0.15
**Body mass index (kg/m** **^2^** **)**	−0.20	0.06
**Sperm morphology (%)**	0.50	p < 0.001 **
**Sperm motility (%)**	0.33	p < 0.001 **
**Sperm count (million/mL)**	0.24	0.02**
**Malondialdehyde (nmol/mL)**	−0.32	p < 0.001 **
**4-Hydroxynonenal (ng/L)**	−0.28	0.01**
**Lipidperoxide (ng/mL)**	−0.09	0.42
**Superoxidedismutase (ng/mL)**	−0.28	0.01**
**Inhibin B (ng/L)**	0.00	0.97
**Total antioxidant capacity (U/mL)**	0.72	<0.01 **

Spearman correlation coefficients (r) between seminal vitamin D and study parameters. shown with two-tailed p-values. Associations with p < 0.05 were considered statistically significant and those with p < 0.001 were considered highly significant.

**Table 3:** Correlation of sperm parameters with oxidative stress markers.

Oxidative stress markers	Morphology (%)		Motility (%)		Sperm count (million/mL)	
	r	p-value	r	p-value	r	p-value
**Malondialdehyde (nmol/mL)**	−0.63	p < 0.001	−0.45	p < 0.001	−0.46	p < 0.001
**4-Hydroxynonenal (ng/L)**	−0.68	p < 0.001	−0.46	p < 0.001	−0.48	p < 0.001
**Lipidperoxide (ng/mL)**	−0.10	0.35	−0.06	0.59	−0.21	0.05
**Superoxidedismutase (ng/mL)**	−0.25	0.02	−0.13	0.24	−0.11	0.29
**Inhibin B (ng/L)**	−0.11	0.29	−0.07	0.52	−0.02	0.82
**Total antioxidant capacity (U/mL)**	0.43	p < 0.001	0.18	0.08	0.21	0.04
**Vitamin D (fmol/L)**	0.50	p < 0.001	0.33	p < 0.001	0.24	0.02

Correlations are Spearman’s *r*. p < 0.05 was considered significant; p < 0.001 was considered highly significant.

**Table 4:** Association between vitamin D levels and study parameters.

Study Parameters	Vitamin D levels			p-value
	Vitamin D deficient	Vitamin D inefficient	Vitamin D sufficient	
**Age (years)**	33.79 ± 4.56	36.16 ± 8.38	36.54 ± 5.86	0.20
**Body mass index (kg/m** **^2^** **)**	27.9 ± 3.73	26.25 ± 3.22	25.68 ± 4.12	0.06
**Sperm morphology (%)**	1.82 ± 4.1	7.12 ± 7.42	9.49 ± 7.55	p < 0.001
**Sperm motility (%)**	6.34 ± 4.51	17.47 ± 19.8	17.21 ± 15.47	0.01
**Sperm count (million/mL)**	10.35 ± 6.87	37.31 ± 39.11	29.92 ± 31.4	p < 0.001
**Malondialdehyde (nmol/mL)**	11.58 ± 5.7	8.38 ± 5.33	7.48 ± 4.35	0.01
**4-Hydroxynonenal (ng/L)**	1.6 ± 0.67	1.02 ± 0.65	1.18 ± 0.81	0.01
**Lipid peroxide (ng/mL)**	62.82 ± 19.76	55.84 ± 17.9	57.79 ± 18.47	0.37
**Superoxide dismutase (ng/mL)**	109.33 ± 51.08	108.11 ± 62.89	81.61 ± 42.04	0.05
**Inhibin B (ng/L)**	41.08 ± 19.34	42.39 ± 30.75	41.14 ± 21.51	0.97
**Total antioxidant capacity (U/mL)**	2.29 ± 0.63	2.83 ± 0.78	3.69 ± 0.79	p < 0.001

Values are mean ± SD. Group comparisons used one-way ANOVA. p < 0.05 was considered significant; p < 0.001 was considered highly significant. Vitamin D categories were defined as deficient (<20 ng/mL), insufficient (20–29 ng/mL), and sufficient (≥30 ng/mL.)
